# Pulmonary Arterial Hypertension and Consecutive Right Heart Failure Lead to Liver Fibrosis

**DOI:** 10.3389/fcvm.2022.862330

**Published:** 2022-03-17

**Authors:** Florian Hamberger, Ekaterina Legchenko, Philippe Chouvarine, Young Seon Mederacke, Richard Taubert, Martin Meier, Danny Jonigk, Georg Hansmann, Ingmar Mederacke

**Affiliations:** ^1^Department of Gastroenterology, Hepatology and Endocrinology, Hannover Medical School, Hannover, Germany; ^2^Department of Pediatric Cardiology and Critical Care, Hannover Medical School, Hannover, Germany; ^3^Laboratory Animal Science, Small Animal Imaging Center, Hannover Medical School, Hannover, Germany; ^4^Institute of Pathology, Hannover Medical School, Hannover, Germany; ^5^Member of the German Center for Lung Research (DZL), Biomedical Research in Endstage and Obstructive Lung Disease Hannover (BREATH), Hannover, Germany

**Keywords:** congestive hepatopathy, pulmonary arterial hypertension, liver fibrosis, hepatic stellate cells, hypoxia

## Abstract

Hepatic congestion occurs in patients with right heart failure and can ultimately lead to liver fibrosis or cardiac cirrhosis. Elevated pulmonary arterial pressure is found in patients with hepatic congestion. However, whether pulmonary arterial hypertension (PAH) can be a cause of liver fibrosis is unknown. The aim of this study was to investigate whether rats in the SuHx model with severe PAH develop liver fibrosis and to explore the mechanisms of congestive hepatic fibrosis both in rats and humans. To achieve this, PAH was induced in six to eight-week old male Sprague Dawley rats by a single subcutaneous injection of the VEGFR 2 inhibitor SU5416 and subsequent hypoxia for 3 weeks, followed by a 6-week period in room air. SuHx-exposed rats developed severe PAH, right ventricular hypertrophy (RVH), and consecutive right ventricular failure. Cardiac magnetic resonance imaging (MRI) and histological analysis revealed that PAH rats developed both hepatic congestion and liver fibrosis. Gene set enrichment analysis (GSEA) of whole liver RNA sequencing data identified a hepatic stellate cell specific gene signature in PAH rats. Consistently, tissue microarray from liver of patients with histological evidence of hepatic congestion and underlying heart disease revealed similar fibrogenic gene expression patterns and signaling pathways. In conclusion, severe PAH with concomitant right heart failure leads to hepatic congestion and liver fibrosis in the SU5416/hypoxia rat PAH model. Patients with PAH should therefore be screened for unrecognized liver fibrosis.

## Introduction

Liver fibrosis or cirrhosis are the long-term consequences of chronic liver injury and do not only occur as a sequelae of primary hepatic diseases such as infectious viral hepatitis or alcoholic liver disease, but can also develop secondary to heart disease (1). While *acute* right or left heart failure with low cardiac output can result in ischemic hepatitis with a rapid increase in aminotransferase levels (2), *chronic* congestive heart failure causes elevated central venous pressure, congestive hepatopathy (3, 4), and eventually liver fibrosis or cardiac cirrhosis (5). The etiology of right heart failure predisposing to hepatic congestion include tricuspid regurgitation, mitral stenosis, cardiomyopathy, constrictive pericarditis, and/or pulmonary hypertension (PH) in its precapillary, postcapillary or combined forms (cor pulmonale) (6–8). Moreover, chronic hepatic congestion after the Fontan procedure (no subpulmonary ventricle) causes liver fibrosis in almost all patients with single ventricle physiology, and up to 40% of those patients present with bridging fibrosis 10 years following Fontan procedure (9). All these conditions lead to elevated right sided filling pressure, but it is not known to what extent pulmonary vascular alterations such as PH, defined as a mean pulmonary artery pressure (mPAP) >20 mmHg (10, 11), predispose or cause hepatic congestion and liver fibrosis. A study investigating hemodynamic alterations in patients with chronic congestion observed an elevated average mPAP of 35 mmHg in patients with chronic hepatic congestion (12). Pulmonary arterial hypertension (PAH) is characterized by obliteration of small pulmonary arteries, increased pulmonary vascular resistance, and isolated precapillary PH with normal left-sided filling pressure. In contrast, one of the major causes of post-capillary PH is left heart disease with elevated left-sided filling pressure. Both HFpEF (heart failure with preserved ejection fraction) and HFrEF (heart failure with reduced ejection fraction) can cause PH (13–16), however, recent studies suggest that PH is more common in patients with HFpEF (38% in HFpEF vs. 22.6% in HFrEF) (17, 18).

Patients with progressive PH may develop right heart failure with elevated right-sided filling pressure, subsequent increase in central venous pressure and potentially congestive hepatopathy (3, 4). However, clinical evaluation for liver fibrosis or cirrhosis in patients with heart or lung disease and biventricular circulation is not routinely performed, although higher liver fibrosis scores are associated with increased risks of all-cause mortality among patients with coronary artery disease (19) or HFpEF (20).

The mechanisms underlying liver fibrosis in right, left or biventricular heart failure are incompletely understood. It has been proposed that congestive cirrhosis is a response to intrahepatic thrombosis (21) and hypercoagulation (22–25). However, there is an unmet need for a small animal model with heart failure and consecutive hepatic fibrosis or cirrhosis to study the underlying mechanisms more in detail (26, 27).

None of the published models on right ventricular (RV) pressure or volume overload studied or even demonstrated that consecutive congestive heart failure—when evident—causes substantial liver fibrosis (27–30). Toxin-induced models of RV hypertrophy or failure such as monocrotaline (27) are suboptimal since they are associated with liver toxicity (31) and myocarditis (32).

The SU5416/hypoxia (SuHx) rat model, i.e., a combination of vascular endothelial growth factor 2 (VEGFR2) blockade and chronic hypoxia, is one of the most established PAH models that can be used to induce PH and subsequent right heart failure in rats (27, 32, 33). The aim of this study was to investigate whether rats in the SuHx model with severe PAH develop liver fibrosis and to explore the mechanisms of such congestive hepatic fibrosis both in rats and humans.

## Materials and Methods

### Study Design: SU5416/Hypoxia Rat PAH Model

All animal experiments were conducted under the approval of the Niedersaechsisches Landesamt für Verbraucherschutz und Lebensmittelsicherheit (LAVES; #15/2022).

**Figure 1:**
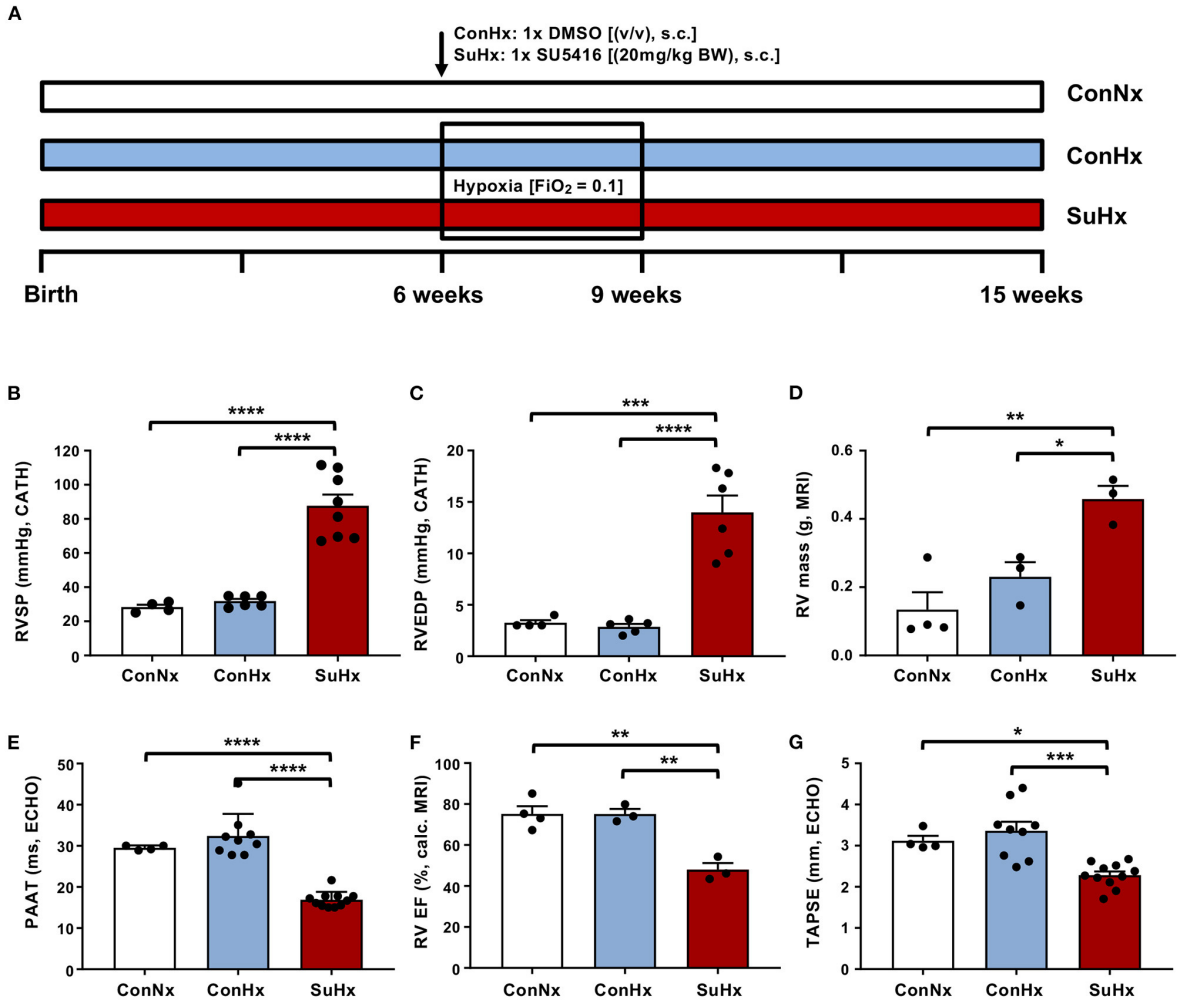
The SU5416/hypoxia model leads to severe PAH in rats. **(A)** Experimental setup. Six- to eight-week-old male Sprague Dawley rats were purchased from Charles River and divided into three different treatment groups: (i) Normoxia (ConNx), (ii) Hypoxia (ConHx) [injected once s.c. with vehicle (DMSO), then exposed to chronic hypoxia (FiO_2_ 0.1) for 3 weeks, followed by a 6-week period in room air (FiO_2_ 0.21)], (iii) SU5416/hypoxia (SuHx) [injected with the VEGFR2 inhibitor SU5416 (Sigma), 20 mg/kg per dose s.c. dissolved in DMSO and subsequently exposed to chronic hypoxia (3 weeks), followed by 6 weeks of room air]. **(B–D)** Right ventricular systolic pressure (RVSP), right ventricular end diastolic pressure (RVEDP), both measured by heart catheterization, and right ventricular (RV) mass, measured by MRI of analyzed rats. **(E–G)** Pulmonary artery acceleration time (PAAT), measured by echocardiography (ECHO), right ventricular ejection fraction (RV EF), calculated from MRI data and tricuspid annular peak systolic excursion (TAPSE), measured by ECHO. Means ± SEM, ConNx *n* = 13, ConHx *n* = 12, SuHx *n* = 7, ANOVA-Bonferroni *post-hoc* test, *p < 0.05, **p < 0.01, ***p < 0.001, ****p < 0.0001.

Six- to eight-week-old male Sprague Dawley rats were purchased from Charles River (Germany), matched by age, and separated into three different treatment groups. The untreated control group (ConNx) was kept in room air (FiO_2_ 0.21) for the whole duration of the experiment (9 weeks). The control/hypoxia group (ConHx) was injected once s.c. with vehicle DMSO, then exposed to chronic hypoxia (FiO_2_ 0.1) for 3 weeks, followed by a 6-week period in room air. The SU5416/hypoxia group (SuHx) was treated with VEGFR2 inhibitor SU5416 (Sigma-Aldrich, St. Louis, MO, USA) (1x SU5416, 20 mg/kg per dose, s.c. dissolved in DMSO) and subsequently exposed to chronic hypoxia (3 weeks), followed by 6 weeks of room air (Figure 1A). At the end of the experiment, when severe PAH and right ventricular (RV) dysfunction were evident, echocardiography (ECHO), cardiac magnetic resonance imaging (MRI), and cardiac catheterization (closed chest) were performed as described previously (33). Subsequently, the animals were sacrificed and organs were harvested for further analysis.

### Immunohistochemical Staining and Microscopy

Tissue from rat livers was collected from the median and left lateral lobes, fixed with 4% paraformaldehyde, embedded in paraffin and cut to yield 2 μm sections. Afterwards the slides were deparaffinized and rehydrated with xylene and graded ethanol. Hematoxylin and Eosin (H&E), Masson's Trichrome and Picrosirius Red stainings were done according to routine protocols. For immunohistochemistry (IHC), antigen retrieval was performed with either citrate buffer (ab93678, Abcam, Cambridge, UK) or Tris-EDTA buffer (ab93684, Abcam, Cambridge, UK), depending on the antibody used. Primary and secondary antibodies were used as follows: CD3 (1:200, ab16669, Abcam, Cambridge, UK), CD19 (1:100, MAB7489, R&D Systems, Minneapolis, MN, USA), CD68 (1:100, MCA341R, Bio-Rad, Hercules, CA, USA), Von Willebrand Factor (1:200, A0082, Agilent Dako, Santa Clara, CA, USA), Fibrin (1:250, MABS2155, Sigma-Aldrich, St. Louis, MO, USA), mouse anti-rabbit IgG-HRP (1:500, sc-2357, Santa Cruz Biotechnology, Dallas, TX, USA), goat anti-mouse IgG-HRP (1:500, sc-2005, Santa Cruz Biotechnology, Dallas, TX, USA). Antibodies were visualized using ImmPACT DAB solution (Vector Laboratories, Burlingame, CA, USA) and counterstained with hematoxylin solution. The stained tissue sections were scanned with an Aperio CS2 (Leica, Wetzlar, Germany) slide scanner and analyzed with ImageJ software (Version 1.51n).

### RNA Isolation

Liver tissue collected for RNA isolation was snap frozen in liquid nitrogen and stored at −80°C. For RNA extraction, the tissue was submerged in TRIzol (Life Technologies, Carlsbad, CA, USA), subjected to a tissue grinder and was further processed according to the TRIzol manufacturer's protocol.

### Reverse Transcription and Quantitative PCR

Reverse transcription was performed with the High-Capacity cDNA Reverse Transcription Kit (Applied Biosystems, Waltham, MA, USA) and qPCR was run on an ABI 7300 Real-Time PCR System (Applied Biosystems, Waltham, MA, USA) using qPCR Master Mix Plus (Eurogentec, Seraing, Belgium) and TaqMan Probes (ThermoFisher Scientific, Waltham, MA, USA). The following primers were used: *Acta2* (Mm01546133_m1), *Col1a1* (Mm00801666_g1), *Timp1* (Mm00441818_m1), *TNFa* (Mm00443258_m1), *Desmin* (Mm00802455_m1), *vWF* (Rn01492158_m1), *CD4* (Rn00562286_m1), *CD8* (Rn00580577_m1), *CD3* (Rn00565890_m1), *CD19* (Rn01507619_g1), *CD68* (Rn01495634_g1), and *18s* (4319413E, ThermoFisher, Waltham, MA, USA) as a housekeeping gene.

### RNA Sequencing and Analysis

Sequencing of mRNA was performed by BGI (Hong Kong). Prior to sequencing, RNA quality was assessed by RIN analysis on a 2100 Bioanalyzer (Agilent, Santa Clara, CA, USA). TruSeq transcriptom libraries were prepared for 3 samples per group following established protocols from Illumina and then sequenced with HiSeq2000 using 90bp paired-end reads. The sequencing reads were aligned to the rat genome (Rnor_6.0.96) using STAR (34) and the read counts corresponding to the Ensembl-annotated genes were identified using RSEM (35). Differential gene expression was analyzed using DEseq (36) after within-lane GC normalization by EDAseq (37). The sharing mode parameter was set to “fit-only” for the estimateDispersions method in DEseq. Benjamini-Hochberg false discovery rate (FDR) procedure was applied to correct for multiple testing. Additionally, the gene filtering procedure developed by Bourgon et al. (38) was applied to improve the detection power. mRNAs with FDR-adjusted *P* < 0.05 were considered significantly differentially expressed. Volcano plots were created in R using the ggplot2 and ggrepel packages. Heatmaps were created in Excel. The gene set enrichment analysis was performed using the pre-ranked analysis option in GSEA (39, 40). The ranking was based on the expression score calculated as Log2(fold change)^*^[-Log10(q-value)] from every gene with a *P* < 0.05. GSEA was run on the complete hallmark gene set collection from the Molecular Signatures Database (MSigDB, h.all.v7.4.symbols.gmt) (41), the hepatic stellate cell signature, quiescent and activated gene sets derived from Zhang et al. (42), and the KEGG pathway gene sets used by the nCounter® Fibrosis Panel (Nanostring Technologies, Seattle, WA, USA), using the Rat_ENSEMBLE_Gene_ID_Human_Orthologs_MSigDB.v7.4.chip platform to apply the “collapse/remap to gene symbols” option. The RNA sequencing data have been deposited with links to BioProject accession number PRJNA 807371 in the NCBI BioProject database (https://www.ncbi.nlm.nih.gov/bioproject/).

### Human Tissue Specimen and nCounter^®^ Fibrosis Panel

We screened patients treated at Hannover Medical School with underlying heart failure and clinical or laboratory suspicion of advanced liver disease who underwent transjugular liver biopsy for further diagnostic. A total of seven patients were identified with the histological diagnosis of hepatic venous congestion and no evidence for viral hepatitis, genetic or metabolic liver disease. As controls, we used five patients with autoimmune hepatitis in clinical remission with absent liver fibrosis. Grading and staging of liver biopsies was performed by Ishak score (43). Liver elastography was determined by acoustic radiation force impulse (ARFI) elastography (44). Detailed patient characteristics are provided in the Supporting Information (Supplementary Table 1). RNA extraction from formalin-fixed paraffin-embedded (FFPE) sections was carried out using the Maxwell® RSC RNA FFPE Kit (Promega, Madison, WI, USA). Of the purified RNA, 200 ng were loaded on the nCounter® Digital Analyzer and analyzed by nSolver software v4.0 with the nCounter® Fibrosis Panel (Nanostring Technologies, Seattle, WA, USA). The study was approved by the Ethics Committees at the Hannover Medical School (No. 3381-2016).

### Statistical Analysis

Statistical analysis was done using Prism version 8 (GraphPad, San Diego, CA) and R 4.0.5 (The R Project for Statistical Computing). Shapiro-Wilk test was used to test for normal distribution. Differences between two groups were calculated by Welch's *t-*test and assessment of differences of multiple groups was performed by one-way ANOVA and Bonferroni's multiple comparisons test. For correlation analysis between hemodynamic data and gene expression, data was tested for normality by multivariate Shapiro test followed by two-tailed Pearson or Spearman correlation, depending on normality. All data are expressed as mean ± SEM.

## Results

### Exposure to SU5416/Hypoxia (SuHx) Leads to Severe, Sustained PAH and RV Failure in Rats

To investigate the effects of severe PAH and RV failure on the development of liver fibrosis, we employed the SU5416/hypoxia rat model (Figure 1A). In this well-accepted PAH model, hypoxia alone (ConHx; +DMSO s.c. = vehicle of SU5416) did not induce sustained pulmonary hypertension or even right heart failure 6 weeks after the end of hypoxia. However, rats exposed to a single injection of SU5416, a VEGFR 2 inhibitor, and subsequent chronic hypoxia for 3 weeks, developed a near-systemic increase of right ventricular systolic pressure (RVSP; Figure 1B), indicating PAH, elevated right ventricular end-diastolic pressure (RVEDP; Figure 1C) as surrogate of RV diastolic dysfunction, and increased RV mass (Figure 1D) indicating RV hypertrophy, compared to animals in the ConNx or ConHx control groups. Moreover, pulmonary artery (PA) acceleration time (PAAT) as an inverse echocardiographic indicator of PA pressure was decreased (Figure 1E). Consequently, RV ejection fraction (RV EF) by MRI (Figure 1F), and tricuspid annular peak systolic excursion by ECHO (TAPSE; Figure 1G), both established markers for RV systolic function, were decreased vs. controls. Taken together these results demonstrate, that SuHx-exposed rats developed severe PAH, right ventricular hypertrophy (RVH), and consecutive right ventricular failure.

### Rats With Severe PAH and RV Failure Develop Hepatic Congestion and Liver Fibrosis

**Figure 2:**
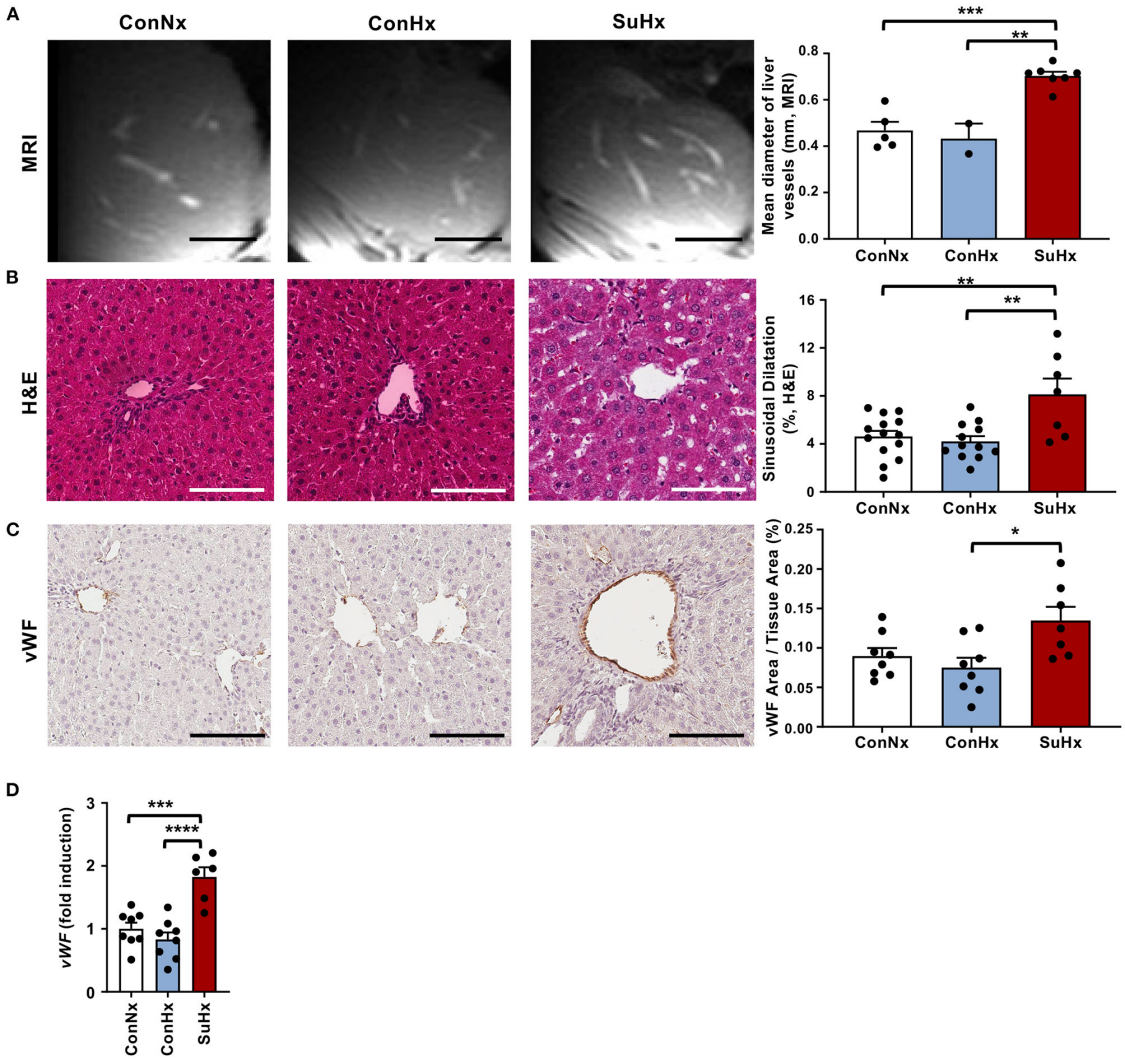
PAH rats show signs of hepatic congestion. **(A)** Representative MRI images and quantification of mean diameter of visible liver vessels in the livers of the different treatment groups. Scale bars 5 mm **(B)** Representative pictures and quantification of vessel area of Hematoxylin and Eosin (H&E) stained livers of analyzed rats. **(C)** Representative pictures and quantification of IHC for vascularization marker von Willebrand factor (*vWF*). **(D)** Relative expression of von Willebrand factor (*vWF*) determined by qPCR from whole livers. Means ± SEM, ConNx *n* = 8–13, ConHx *n* = 8–12, SuHx *n* = 7, ANOVA-Bonferroni *post-hoc* test, *p < 0.05, **p < 0.01, ***p < 0.001, ****p < 0.0001, Scale bars 100 μm.

**Figure 3:**
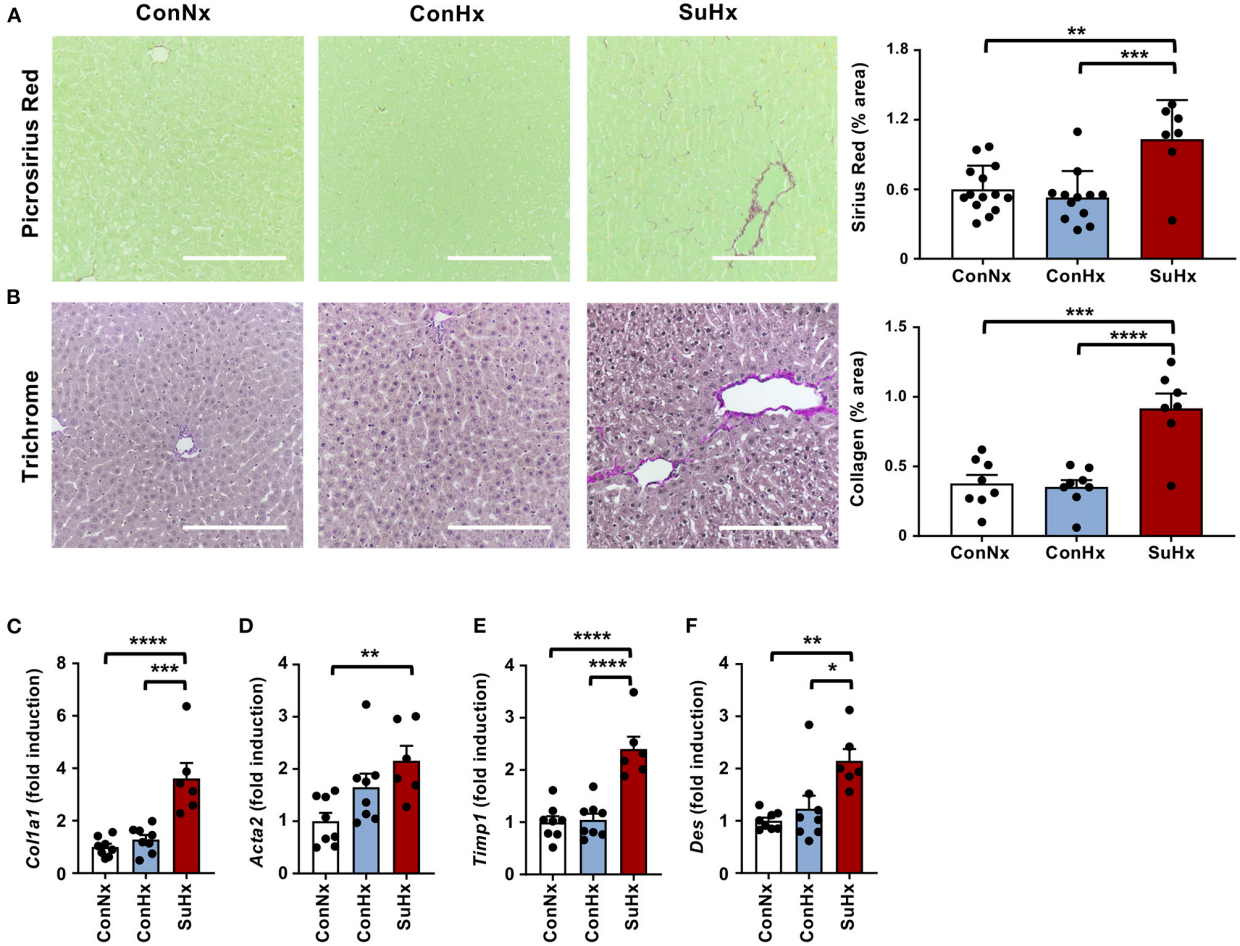
PAH leads to increased liver fibrosis in the SU5416/hypoxia model. **(A)** Representative pictures and quantification of picrosirius red stained livers of the analyzed rats. ConNx *n* = 13, ConHx *n* = 12, SuHx *n* = 7. **(B)** Representative pictures and quantification of Masson's trichrome staining. **(C–F)** Relative expression of fibrogenic markers collagen 1a1 (*Col1a1*), alpha smooth muscle actin (*Acta2*), TIMP metallopeptidase inhibitor 1 (*Timp1)* and desmin (*Des*) determined by qPCR from whole livers. Means ± SEM, ConNx *n* = 8, ConHx *n* = 8, SuHx *n* = 7, ANOVA-Bonferroni *post-hoc* test, *p < 0.05, **p < 0.01, ***p < 0.001, ****p < 0.0001, Scale bars: 100 μm.

Next, we explored whether severe PAH and systolic RV dysfunction may lead to hepatic congestion. We therefore analyzed MRI scans and observed a significantly increased mean diameter of hepatic veins in rats with PAH (Figure 2A). To evaluate whether congestive vessel dilatation is also observed on the microscopic level, we performed H&E staining and found that rats from the SuHx group with severe PAH also showed a significant increase in sinusoidal dilatation (Figure 2B). In line with these results, immunohistochemical staining (IHC) and qPCR analysis revealed increased expression of von Willebrand Factor (*vWF*), a glycoprotein secreted by endothelial cells to mediate platelet adhesion, and associated with thrombosis and liver fibrosis, in the SuHx vs. controls (Figures 2C,D). To assess whether animals with PAH also develop liver fibrosis under these conditions, we performed picrosirius red and Masson's trichrome staining and observed an increase of fibrotic area by 72 and 142%, respectively, in SuHx treated rats (Figures 3A,B) vs. controls. To confirm these results, we performed quantitative real-time PCR analysis and unraveled an increased expression of profibrotic genes, including collagen 1a1 (*Col1a1*), alpha smooth muscle actin (*Acta2*), tissue inhibitor of metalloproteinase metallopeptidase inhibitor 1 (*Timp1*), and desmin (*Des*) (Figures 3C–F). To rule out that SU5416 alone - without exposure to chronic hypoxia - causes liver fibrosis, we treated six to eight-week old male Sprague Dawley rats with SU5416 or vehicle control (DMSO) and kept these animals at normoxia for 9 weeks. There was no significant difference in liver fibrosis and inflammation between both groups (Supplementary Figures 1, 2), excluding VEGFR2 inhibition as a possible confounder or cause of liver fibrosis in our model.

### Microthrombosis and Inflammation Are Evident in the Congested Livers of PAH Rats

**Figure 4:**
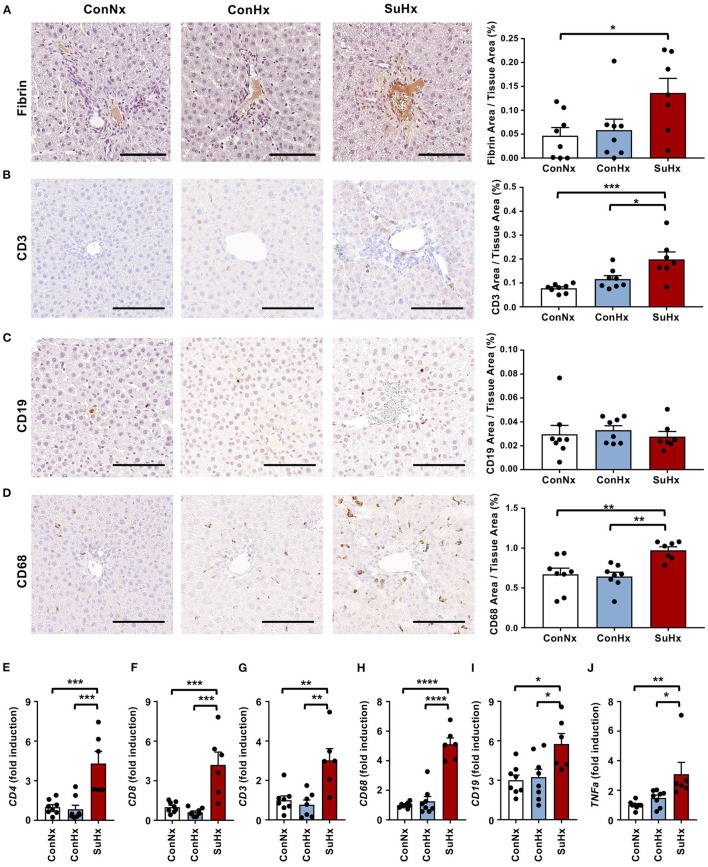
PAH livers show increased coagulation and liver immune cell infiltration is mainly comprised of T-cells and macrophages. **(A–D)** Representative pictures and quantification of IHC for coagulation marker fibrin, as well as immune cell markers of T-cells (CD3), B-cells (CD19) and macrophages (CD68). **(E–J)** Relative expression of immune cell markers CD4, CD8, CD3, CD68, CD19 and TNFa determined by qPCR from whole liver. Means ± SEM, ConNx *n* = 8, ConHx *n* = 8, SuHx *n* = 7, ANOVA-Bonferroni *post-hoc* test, *p < 0.05, **p < 0.01, ***p < 0.001, ****p < 0.0001, Scale bars: 100 μm.

To investigate whether inflammation and intrahepatic thrombosis play a role in the development of liver fibrosis in rats with PAH, we next performed IHC for the coagulatory marker fibrin and the immune cell markers CD3 (T-cells), CD19 (B-cells) and CD68 (macrophages). SuHx rats showed significantly increased fibrin deposition in the liver vessels (Figure 4A), as well as increased numbers of CD3 and CD68 positive cells compared to the control groups, while CD19 staining showed no significant difference between the three groups (Figures 4B–D). Validation by qPCR revealed a markedly increased expression of CD4 and CD8 T cells as well as macrophage marker CD68 whereas CD19 and TNFα showed a lower yet still significant increase in the SuHx group compared to controls (Figures 4E–J).

### Severe PAH and RV Dysfunction Are Associated With a Proinflammatory Hepatic Gene Program and a Hepatic Stellate Cell-Specific Gene Signature in the Liver

**Figure 5:**
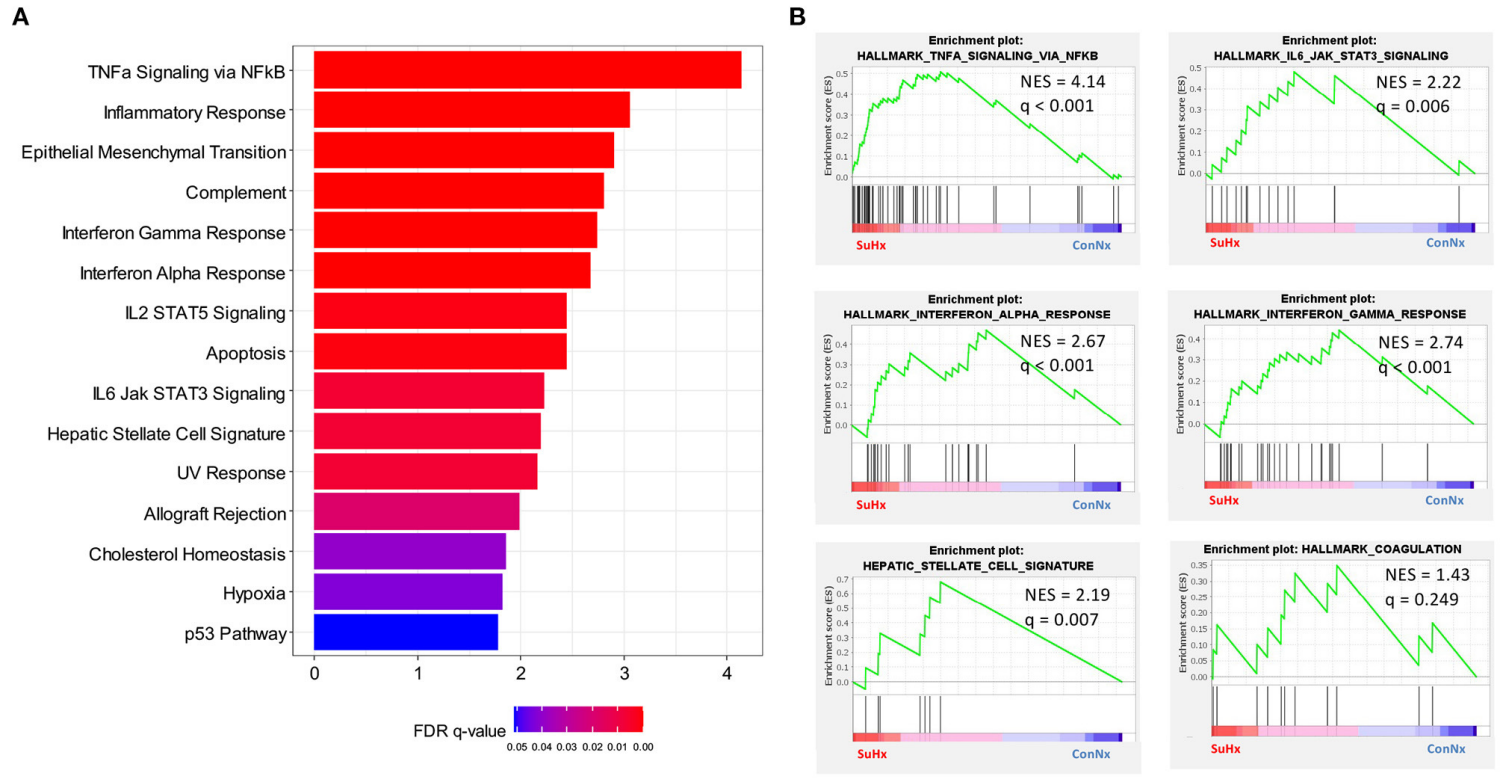
RNA Sequencing reveals inflammatory and fibrotic gene expression in livers of PAH rats. **(A)** GSEA analysis of differentially regulated genes in SuHx treated rats compared with ConNx control group. Shown are all pathways with FDR adjusted q-values < 0.05. **(B)** Enrichment plots of select pathways from the GSEA analysis. ConNx *n* = 3, SuHx *n* = 3.

To confirm the results of IHC and qPCR, we performed whole liver RNA sequencing on animals of each treatment group (ConNx, ConHx, SuHx) (Supplementary Figure 3). Gene Set Enrichment Analysis (GSEA) showed an enrichment of pathways associated with inflammation and liver fibrosis in SuHx animals, in particular TNFα signaling, IL6-JAK-STAT3 signaling, Interferon alpha (IFNα) and gamma (IFNγ) response (Figures 5A,B). Additionally, in accordance with the results of the fibrin staining, we found the Hallmark Coagulation pathway slightly enriched in SuHx compared to ConNx, although the FDR adjusted *p*-value in this case was not significant (Figure 5B). Lastly, we detected an enrichment of a hepatic stellate cell (HSC) specific gene signature (42), indicating that activation of HSC, the major fibrogenic cell population in the liver, contributes to the observed liver fibrosis.

### Patients With Hepatic Congestion but No Clinically Evident Liver Disease Display Fibrotic Gene Expression Patterns in the Liver

**Figure 6:**
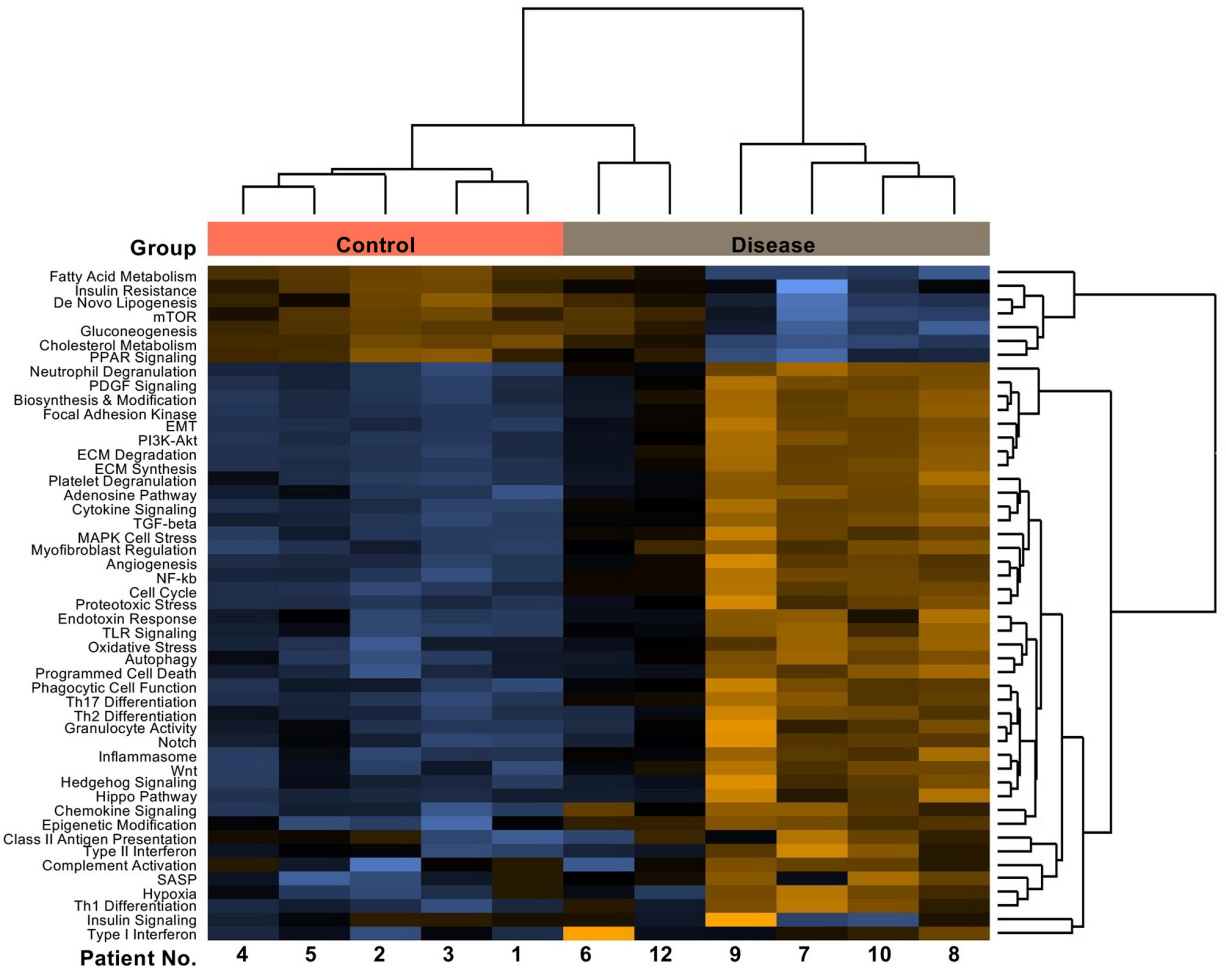
Patients with underlying heart/lung disease display upregulated fibrotic pathways in liver biopsies. Pathway score heatmap of fibrogenic gene sets detected via nCounter® Fibrosis Panel in RNA isolated from FFPE tissues of patients with confirmed histological diagnosis of congestive hepatopathy and underlying heart disease (Disease) and patients with autoimmune hepatitis in remission as control (Control).

**Figure 7:**
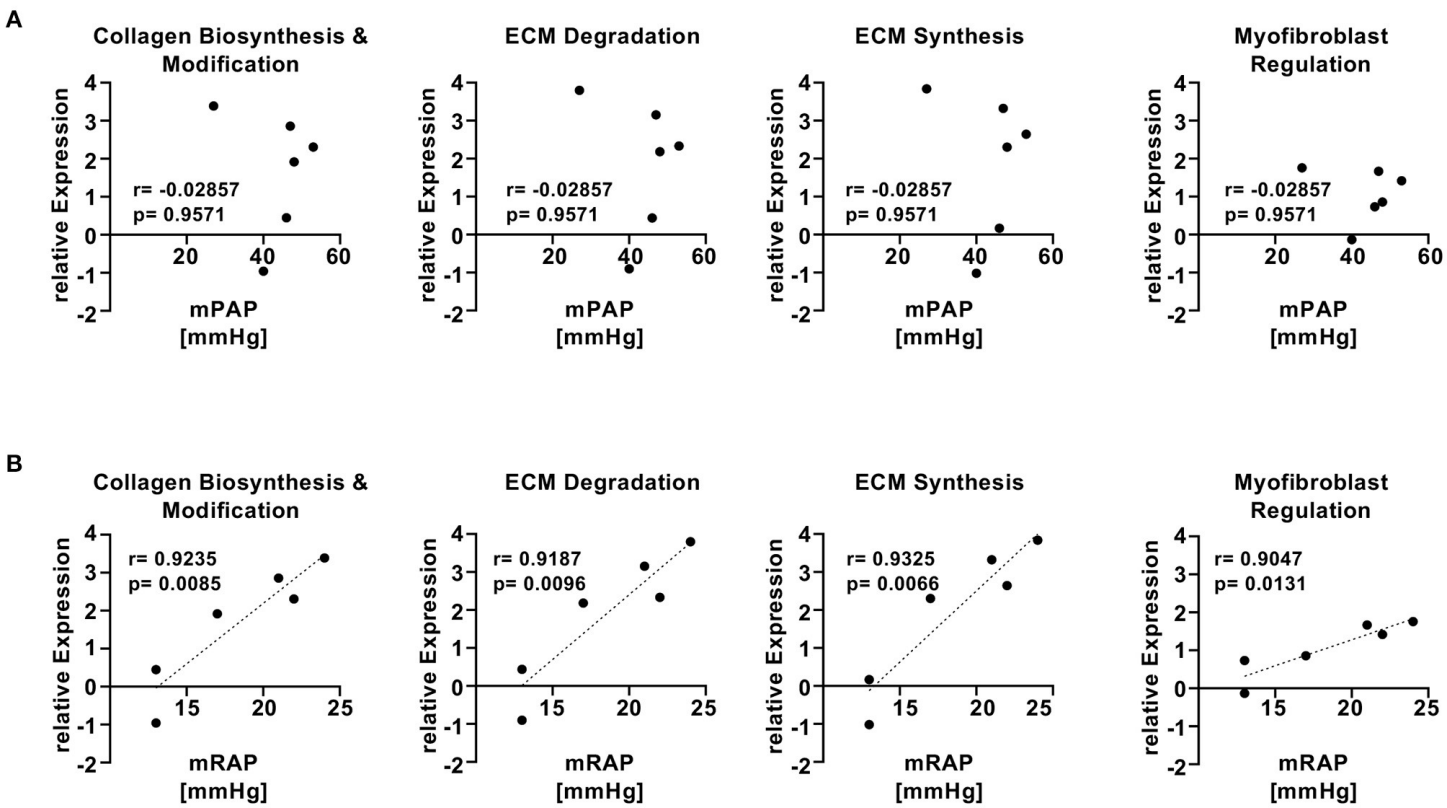
mRAP but not mPAP of patients with hepatic congestion and underlying heart/lung disease shows significant correlation with fibrogenic gene pathways. **(A)** Two-tailed Spearman correlation plots of mean pulmonary arterial pressure (mPAP) vs. relative expression of different fibrogenic pathways from Nanostring analysis. **(B)** Two-tailed Pearson correlation plots of mean right atrial pressure (mRAP) vs. relative expression of different fibrogenic pathways from Nanostring analysis.

**Figure 8:**
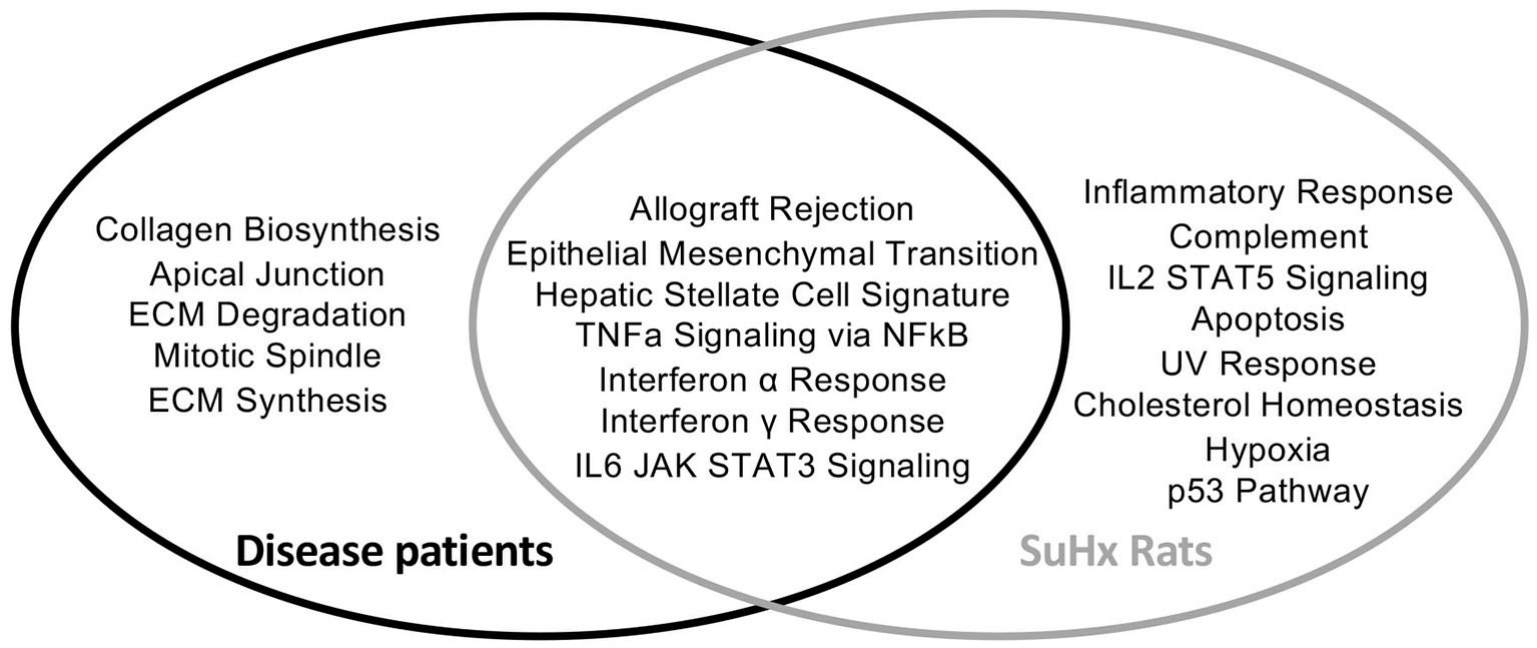
Liver biopsies of patients with underlying heart/lung disease and PAH rats show similarities in upregulated fibrotic pathways. Venn diagram of differentially regulated pathways in disease patients and SuHx treated rats compared to their respective controls. Disease patients *n* = 6, SuHx Rats *n* = 3.

To assess whether the findings in the SU5416/hypoxia rat model of PAH/RV failure could have clinical relevance, we analyzed patients with confirmed histological diagnosis of congestive hepatopathy and underlying heart/lung disease. Detailed patient characteristics are shown in Supplementary Table 1. Patients with autoimmune hepatitis in biochemical and histological remission without evidence for liver fibrosis (Ishak F0) were used as controls (43). Liver function test showed no significant differences in ALT or AST levels, but a significant elevation of AP and gGT in patients with heart failure and hepatic congestion (Supplementary Figure 4). Next, we performed gene expression analysis of liver biopsies using nCounter® Fibrosis Panel (Nanostring Technologies, Seattle, WA, USA). At first, we performed an analysis using the Nanostring nSolver software and revealed an induction of pathways associated with liver fibrosis (Figure 6). When correlating the relative expression of different fibrogenic pathways with mean pulmonary arterial pressure (mPAP) or mean right atrial pressure (mRAP), we observed a significant positive correlation with mRAP while mPAP did not show any association (Figures 7A,B). In order to compare the gene expression patterns of patients with both heart failure and hepatic congestion with the SuHx rat model, we performed GSEA and compared the signaling pathways that are activated in both models (Figure 8). Importantly, a total of seven out of 12 significantly enriched signaling pathways were evident in both SuHx rats and patients with congestive hepatopathy: The enriched pathways included proinflammatory pathways such as tumor necrosis factor alpha and interferon alpha and gamma signaling and a hepatic stellate cell gene signature (42), indicating that HSC are the fibrogenic cell type responsible for the development of liver fibrosis in hepatic congestion in rats and human.

## Discussion

The development of liver fibrosis or cirrhosis in patients with congestive heart disease and heart failure is well-known (10, 19–21, 45–51). However, an adequate *in vivo* model recapitulating both, heart failure and liver fibrosis which would allow to study therapeutic interventions in the heart or lung as well as in the liver is lacking. A previous study using a murine model of pulmonary stenosis observed a higher liver to body-weight ratio in animals with severe pulmonary stenosis (28), but liver fibrosis was not evaluated. Another study applied a model of congestive hepatopathy through partial ligation of the inferior vena cava and showed that chronic hepatic congestion leads to sinusoidal thrombosis and mechanical forces resulting in liver fibrosis (29). However, in none of the aforementioned models heart failure was shown to be the cause liver fibrosis.

Here, we studied the development of liver fibrosis in a rat model of PAH-driven right heart failure (33, 52). We also performed gene expression analysis both in the *in vivo* rat model as well as in liver biopsies from patients with histological diagnosis of hepatic congestion secondary to heart failure. Using the rodent model and human samples, we made several important findings and conclude: (1) rats with severe PAH develop hepatic congestion and liver fibrosis; (2) severe PAH induces hepatic inflammation and microthrombosis; (3) heart failure patients with hepatic congestion but no clinical evidence for underlying liver disease display a fibrotic gene expression pattern in the liver; (4) similar fibrogenic pathways are activated in SuHx-exposed PAH rats on the one hand and patients with hepatic congestion on the other; (5) gene expression data indicates that HSC contribute to the development of liver fibrosis both in PAH rats and in patients with hepatic congestion.

Multiple small animal models of right (27, 53) or left heart failure (26) have been published over the last decades but in none of these models, presence and development of hepatic fibrosis has been systematically studied. Here, we used the SU5416/hypoxia rat model (33, 52) as it features severe PAH and right heart failure (33). In our study, cardiac catheterization, echocardiography and MRI confirmed that animals treated once with the VEGFR2 inhibitor SU5416, followed by subsequent hypoxia for 3 weeks, developed severe PAH and right heart failure. Notably, animals with PAH and RV failure, as judged by greatly increased systolic and end-diastolic RV (filling) pressures, also developed liver fibrosis vs. controls. We propose that the congestion detected in MRI scans and the sinusoidal dilatation observed in liver histology, and the increased fibrin deposits in the liver vessels and immune cell infiltration, likely represent the pathobiological mechanisms of liver fibrosis. Additional mechanisms have been suggested by another group that studied liver fibrosis development in IVC ligated mice; the authors identified mechanosensitive signals released by liver sinusoidal endothelial cells which promoted portal hypertension by recruiting sinusoidal neutrophils and promoting formation of neutrophil extracellular traps and microthrombi (30).

In our study, liver tissue RNA sequencing revealed an altered cholesterol metabolism compared to controls, in addition with major mechanisms others postulated for development of liver fibrosis in congestive heart failure: hypoxic cell death, caused by insufficient arterial perfusion or the generation of microthrombi, triggering hepatic inflammation resulting in activation of fibroblasts, deposition of extracellular matrix, and finally liver fibrosis (21, 29, 50).

The detection of fibrin deposits and expression of vWF suggested thrombosis as another mechanism of liver fibrosis, which has been described by others (29). Furthermore, GSEA showed enrichment of different immune pathways in SuHx livers: increased IFNγ and TNFα signaling point to NK cell activity (54) and that likely induced the observed IL6 and IL2 signaling by macrophages, T cells and fibroblasts detected in IHC stainings of the SuHx livers (55, 56).

Moreover, we found activation of hepatic stellate cells (HSC), the primary precursors to myofibroblasts in liver fibrosis (57), by detecting a signature gene set derived from the findings of Zhang et al. (42). Although alterations in cholesterol metabolism in the liver has not been commonly described in congestive heart failure, it is a major driving factor of fibrosis in non-alcoholic fatty liver disease (NAFLD) (58, 59).

To validate the clinical relevance of the SU5416/hypoxia model, we performed a fibrosis tissue microarray of liver biopsies from patients with hepatic congestion and underlying heart disease and compared the results with the RNA sequencing data from the rat model. Four of the six patients with heart failure and very high mean right atrial pressure (mRAP) (17–24 mmHg) showed markedly enhanced fibrotic gene expression compared to the control patients, while gene expression of the other two patients (#6 and #12) with the lowest mRAP had a RNA expression profile similar to controls, indicating that mRAP drives hepatic congestion and consecutive liver fibrosis. Laboratory diagnostic revealed significant elevation of alkaline phosphatase (AP) and gamma-glutamyl transferase (gGT) in patients with hepatic congestion, while there was no difference in aminotransferases between patients and controls. The elevation of AP and gGT indicates congestive injury rather than ischemic hepatic injury (4, 60). In line with these results, liver stiffness values of heart failure patients were elevated indicating increased liver stiffness caused by hepatic congestion (61).

Our study has several limitations. The degree of fibrosis in the examined animals was comparatively mild, compared to the IVC ligation model published by Simonetto et al. (29), but it has to be considered that IVC ligation is a non-physiological strong congestion, that rather imitates acute Budd Chiari Syndrome than congestive hepatopathy. Moreover, liver fibrosis in humans develops over years and decades and our rodent model covered only several weeks. It is therefore likely, that a longer duration of the SuHx rat experiment could have led to more severe liver fibrosis.

In future studies, it will be important to explore whether moderate or advanced liver fibrosis is common in PAH patients, patients with HFpEF, and those with heart failure and combined pre- and postcapillary pulmonary hypertension (CpcPH) since in these patients, liver fibrosis possibly represents an unrecognized risk factor relevant for mid to long-term outcome, including non-cardiopulmonary surgical or interventional procedures. Accordingly, when patients with end stage heart or liver disease are evaluated for heart and/or lung transplantation, evaluation of liver fibrosis is mandatory (5, 62, 63), because liver cirrhosis is associated with increased mortality thereafter (63, 64). Therefore, advanced liver fibrosis is an exclusion criterion for heart transplantation in patients with Ebstein's malformation or those with single ventricles and a Fontan circulation (45, 46). In these patients, the underlying liver disease may require simultaneous heart-liver transplantation in expert centers (65).

In conclusion, our study demonstrates that severe PAH with concomitant right heart failure leads to liver fibrosis in the SU5416/hypoxia PAH rat model. We also identified a HSC specific gene signature in PAH rats that was recapitulated in liver of patients with histological evidence of hepatic congestion and underlying heart disease, indicating HSC being involved in the increase in liver fibrosis. No liver tissue from patients with different severity of PAH or Fontan patients was available, so that the validity of our findings in these patients needs to be recapitulated. However, our results suggest that it could be beneficial to recognize liver fibrosis in patients with PAH.

Therefore, we propose that patients with PAH should be screened for unrecognized liver fibrosis.

## Data Availability Statement

The RNA sequencing data have been deposited with links to BioProject accession number PRJNA 807371 in the NCBI BioProject database (https://www.ncbi.nlm.nih.gov/bioproject/).

## Ethics Statement

The studies involving human participants were reviewed and approved by the Ethics Committees at the Hannover Medical School (No. 3381-2016). The patients/participants provided their written informed consent to participate in this study. The animal study was reviewed and approved by Niedersaechsisches Landesamt für Verbraucherschutz und Lebensmittelsicherheit (LAVES; #15/2022).

## Author Contributions

FH performed experiments, analyzed data, performed statistical analyses, and drafted the manuscript. EL performed animal experiments, analyzed data, and critically reviewed the manuscript. PC performed the bioinformatics analysis and critically reviewed the manuscript. YSM collected patient data, contributed to data analysis, and critically reviewed the manuscript. RT provided patients samples and critically reviewed the manuscript. MM performed and analyzed MRI imaging. DJ performed experiments, analyzed data, and critically reviewed the manuscript. GH and IM designed and supervised the study, oversaw the statistical analysis, drafted the manuscript, and generated funding. FH, GH, and IM had unrestricted access to all data. All authors contributed to the article and approved the submitted version.

## Funding

IM was supported by a grant from the German Research Foundation (DFG, ME 3723/2-1) and the Lower Saxony Ministry for Science and Culture (REBIRTH Innovation-/Synergy Grant). GH received research grants from the German Research Foundation (DFG KFO311; HA4348/6-2), the European Pediatric Pulmonary Vascular Disease Network (www.pvdnetwork.org), and the Federal Ministry of Education and Research (BMBF 01KC2001B; ViP+ program 03VP08053). YSM was supported by the Young Faculty program of the Hannover Medical School. RT received funding from the German Research Foundation (KFO250) and the CORE100Pilot–Advanced Clinician Scientist program. DJ received a research grant from the German Research Foundation (DFG KFO311; project Z2 to DJ).

## Conflict of Interest

The authors declare that the research was conducted in the absence of any commercial or financial relationships that could be construed as a potential conflict of interest.

## Publisher's Note

All claims expressed in this article are solely those of the authors and do not necessarily represent those of their affiliated organizations, or those of the publisher, the editors and the reviewers. Any product that may be evaluated in this article, or claim that may be made by its manufacturer, is not guaranteed or endorsed by the publisher.
