# Supplementary material for: Pulmonary Arterial Hypertension and Consecutive Right Heart Failure Lead to Liver Fibrosis
Source: Front Cardiovasc Med. 2022 Mar 17;9:862330. doi: 10.3389/fcvm.2022.862330 (PMC8968099; doi:10.3389/fcvm.2022.862330)

**Supplementary Material for**

**Pulmonary arterial hypertension and consecutive right heart failure lead to liver fibrosis**

Florian Hamberger*, Ekaterina Legchenko*, Philippe Chouvarine, Young-Seon Mederacke, Richard Taubert, Martin Meier, Danny Jonigk, Georg Hansmann^#^, Ingmar Mederacke^#^

^#^Corresponding authors. Email: georg.hansmann@gmail.com, mederacke.ingmar@mh-hannover.de

**Contents**

Supplementary Methods

Supplementary Figures S1-S4

Supplementary Table S1

**Supplementary Methods**

**SU5416/normoxia control rats**

Six- to eight-week-old male Sprague Dawley rats were purchased from Charles River (Germany) and separated into two different treatment groups. The vehicle/normoxia group (VehNx) was injected once s.c. with vehicle DMSO, while the SU5416/normoxia group (SuNx) was treated with VEGFR 2 inhibitor SU5416 (Sigma) (1x SU5416, 20 mg/kg per dose, s.c. dissolved in DMSO). Both groups were kept in room air (FiO_2_ 0.21) for 9 weeks, after which the animals were sacrificed and organs were harvested for subsequent analysis.


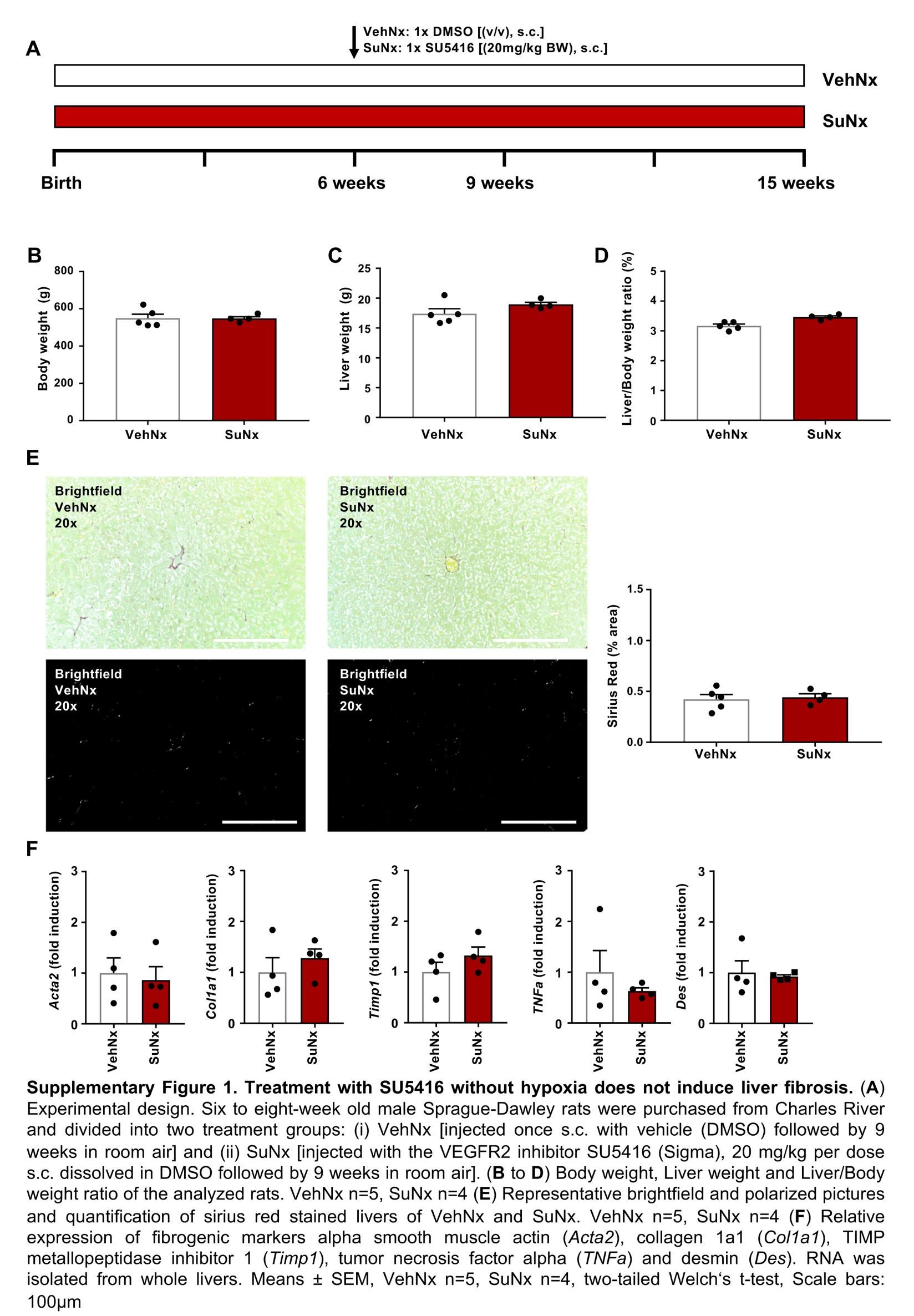


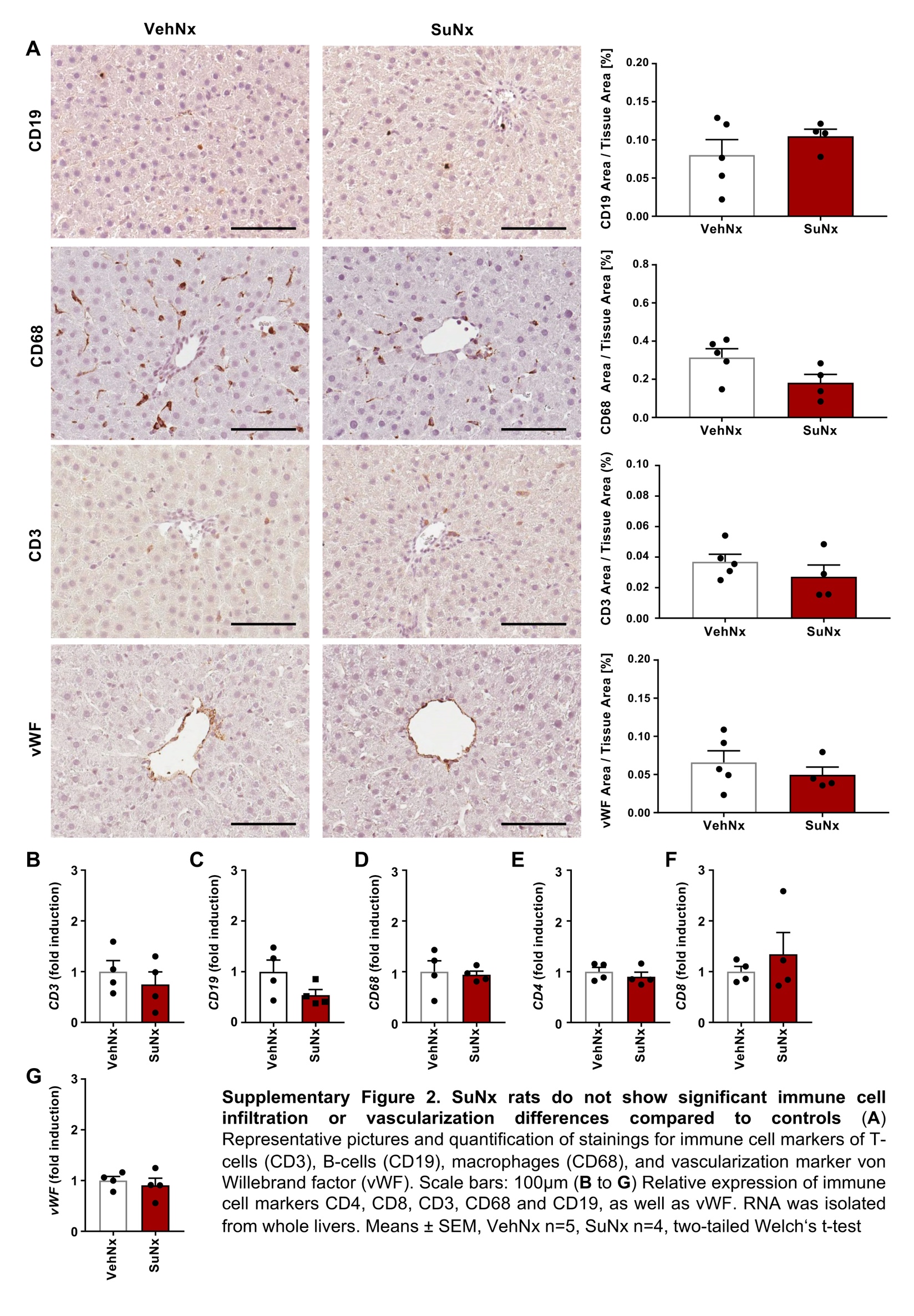


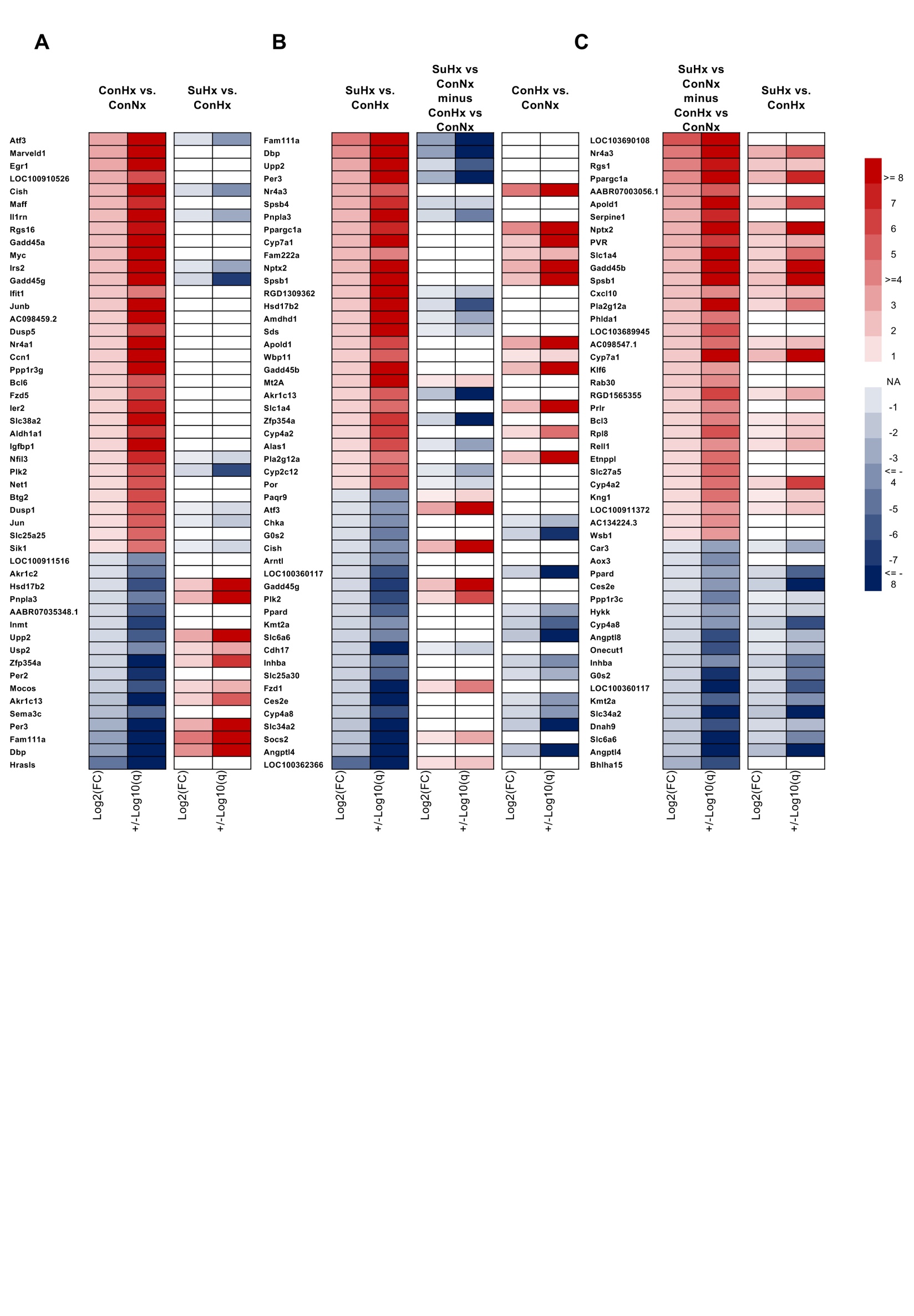


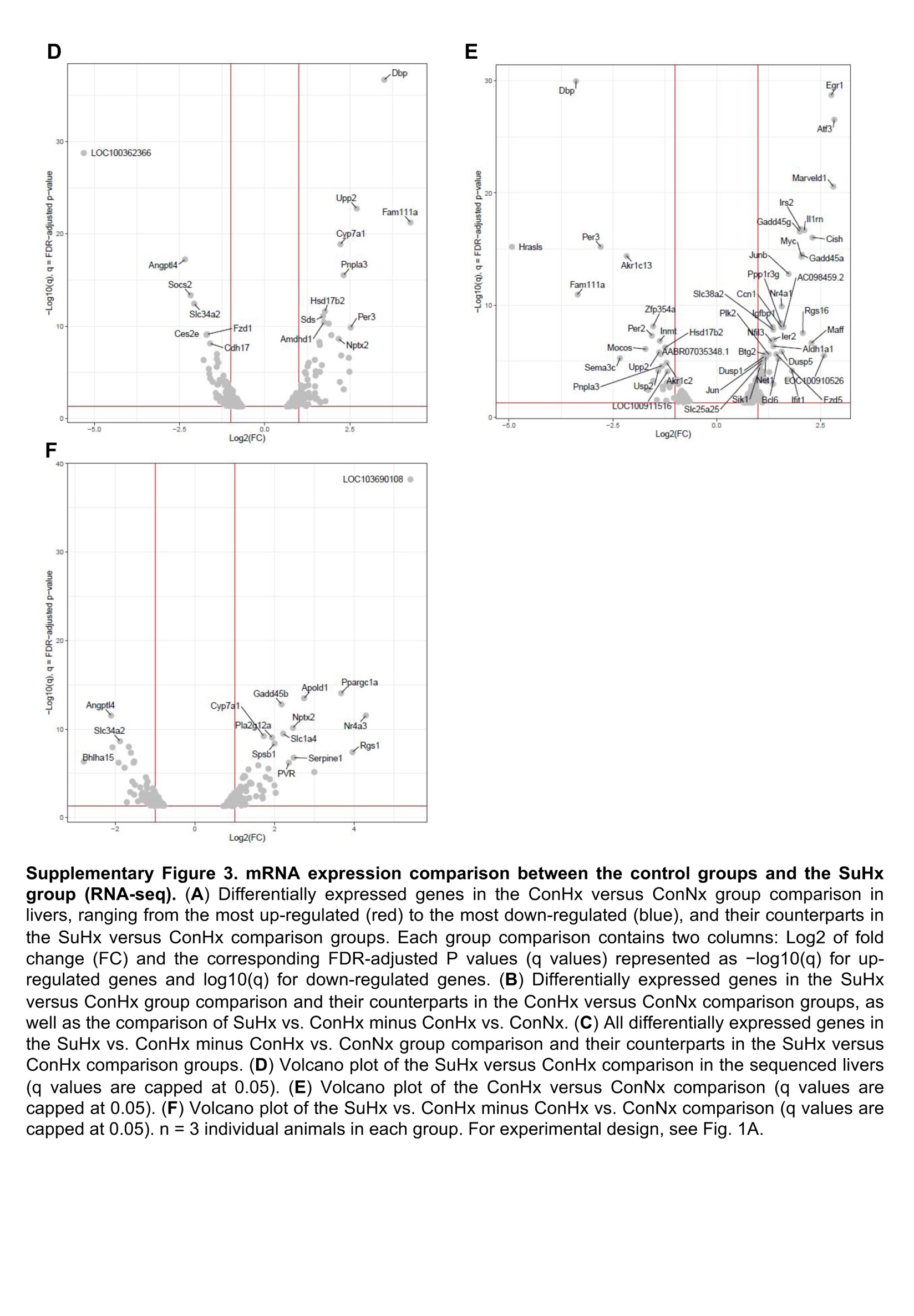


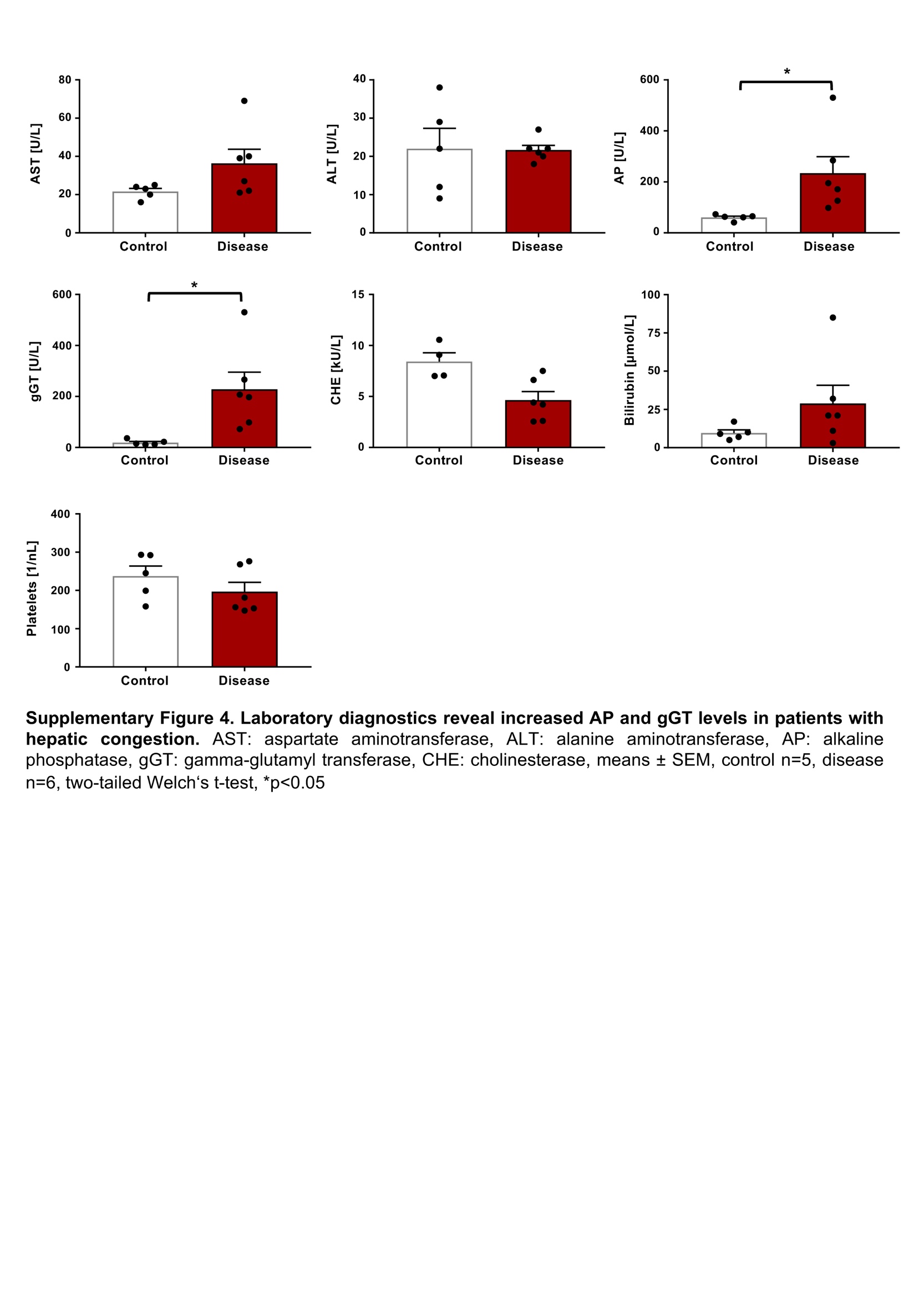

Supplement: Supplementary file 1 [file Table_1.DOCX]
